# Factors influencing the performance of organocatalysts immobilised on solid supports: A review

**DOI:** 10.3762/bjoc.20.183

**Published:** 2024-08-26

**Authors:** Zsuzsanna Fehér, Dóra Richter, Gyula Dargó, József Kupai

**Affiliations:** 1 Department of Organic Chemistry and Technology, Budapest University of Technology and Economics, Műegyetem rkp. 3., H-1111 Budapest, Hungary

**Keywords:** asymmetric synthesis, catalyst recycling, heterogenisation, organocatalysis, solid support

## Abstract

Organocatalysis has become a powerful tool in synthetic chemistry, providing a cost-effective alternative to traditional catalytic methods. The immobilisation of organocatalysts offers the potential to increase catalyst reusability and efficiency in organic reactions. This article reviews the key parameters that influence the effectiveness of immobilised organocatalysts, including the type of support, immobilisation techniques and the resulting interactions. In addition, the influence of these factors on catalytic activity, selectivity and recyclability is discussed, providing an insight into optimising the performance of immobilised organocatalysts for practical applications in organic chemistry.

## Introduction

Organocatalysts are small molecules that do not contain a metal atom in the reaction centre and are able to increase the speed of reactions. They have proven their place among the efficient and robust catalysts on numerous occasions since the two seminal works [1–2] published in 2000. Since then, organocatalysis has been combined with many other areas of research, such as photocatalysis, electrochemistry and mechanochemistry [3–5], while List and MacMillan were awarded the Nobel Prize in 2021 for the development of asymmetric organocatalysis [6]. To date, industrial companies have used a number of asymmetric organocatalytic processes to synthesise pharmaceuticals and fine chemicals on large scales [7].

Catalyst recycling is key from both an economic and an environmental perspective. An efficient catalytic process is characterised by the fact that the catalyst can be easily and, if possible, completely separated from the reaction mixture. Catalysts can be classified into homogeneous and heterogeneous catalysts. In homogeneous catalysis, the reaction components and the catalyst are in the same phase. Active catalytic sites are readily accessible to the reactants and therefore generally result in higher catalytic activity and selectivity [8]. As a result, homogeneous catalysis is generally preferred to heterogeneous catalysis, especially in the fine chemical and pharmaceutical industries [9]. The limitation of homogeneous catalysts, however, is their complex, time-consuming and energy-intensive recovery and subsequent recycling. Therefore, synthetic modification of catalysts is a commonly used method to aid their recovery.

Obstacles to the recycling of homogeneous catalysts can be addressed by heterogenisation of homogeneous catalysts [10], either following their application as homogeneous catalysts or before their application (heterogeneous catalysis). In heterogeneous catalysis, catalysts and reactants are present in different phases. Heterogeneous catalysts are easy to handle and can be easily separated from the reaction mixture by filtration, centrifugation or magnetic force, thus allowing catalysts to be recycled multiple times. Most highly stable and recyclable catalysts are attached to solid supports [11].

Solid supports are generally insoluble materials preferably with a large surface area to maximise the number of active sites for catalyst attachment. The advantages of such supports have been demonstrated by numerous reports on immobilised catalysts [12–14]. The type of support and the immobilisation technique have a major influence on the properties and thus performance of the resulting heterogeneous catalyst.

However, immobilisation and structural modification introduce additional steps in the synthesis of the catalyst. Moreover, the catalytic activity and selectivity of immobilised catalysts are often lower than those of the corresponding native catalysts. In addition, inactivation due to degradation may also occur. For long-term use, consistently high yields (and selectivity) are required over repeated runs, as these are indicative of the robust nature of the catalyst system.

A real challenge is to develop a supported organocatalyst whose catalytic efficiency can be reproduced over a sufficient number of reaction cycles. Despite the difficulty of the challenge, the design of heterogeneous, recyclable organocatalytic systems is of high interest [8]. The continued development of efficient catalytic recovery methods, such as the application of immobilised organocatalysts [14–15] and heterogeneous organocatalysis [16–18], could be a potential driver for the introduction of, for example, enantioselective organocatalysis in the pharmaceutical industry [19]. Knowledge of the factors that influence catalyst performance is crucial to the development of high performance immobilised organocatalysts.

## Review

### Characteristics of the solid support

Considering the support type, organic polymer-supported, silica-supported [20–25], glass beads [26] and magnetic nanoparticle-supported [27–32] organocatalysts are pivotal in the field of immobilised organocatalysts. Polymer-supported organocatalysts are commonly immobilised on polystyrene (PS) [28,33–38], as well as on other materials such as nylon 6,6 [15], chitosan [39–40], and polymethylhydrosiloxane (PMHS) [41]. The role of the polymers as supports for catalysts is not merely passive. These supports significantly influence the reaction environment and catalytic efficiency [42]. Attachment methods, spacer lengths, and polymer nature profoundly impact the catalyst's performance and recyclability. Various immobilization strategies, including covalent bonding and encapsulation, cater to different polymer types. Soluble polymers enhance diffusion, while insoluble ones ensure stability and high loading capacities [42]. Silica is also widely applied due to its ease of functionalisation and thermal stability [43]. The controllability of surface, geometry, and pore size makes silica-based materials sustainable and functionalisable supports for organocatalytic reactions [44].

**Scheme 1:**
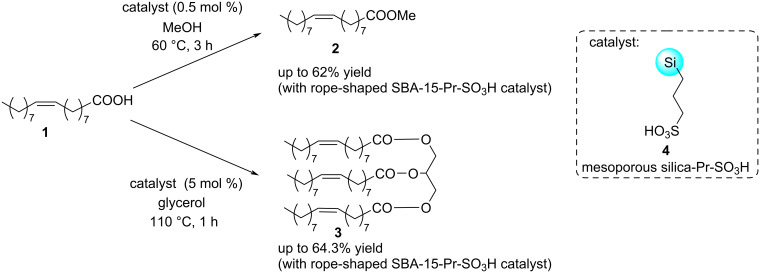
Esterification of oleic acid (**1**) with propylsulfonic acid (Pr-SO_3_H)-functionalised mesoporous silica catalyst **4**.

The particle morphology of mesoporous silica can be tuned to various shapes, including spheres, tubes, and rods of various dimensions [45], by using a co-condensation method performed under low surfactant concentration conditions [46–50], changing the concentration, molecular size, and hydrophilicity/hydrophobicity of the organoalkoxysilane precursors [51]. Various morphologies of mesoporous silica, including fibre, platelet, rod, and film, can be generated by altering the reaction conditions during synthesis [52–57] or by using potassium chloride or ammonium fluoride salts as additives [58–60]. In a comprehensive study [61], the catalytic properties of three types (rope, rod and fibre) of mesoporous silica Santa Barbara Amorphous (SBA-15) and small pore-sized Mobil Composition of Matter (MCM-41) were applied and compared as supports of an organocatalyst. These silicas were modified by incorporating an organosulfonic acid group (propylenesulfonic acid) through a post-synthesis grafting method. Their catalytic performance was studied and compared in the esterification of methanol or glycerol with oleic acid (**1**). It was observed that substrate conversion and product yield also depended on the particle morphology. Rope-type propylsulfonic SBA-15 mesoporous silica gel showed the highest catalytic activity in both studied esterification reactions (Scheme 1).

**Scheme 2:**
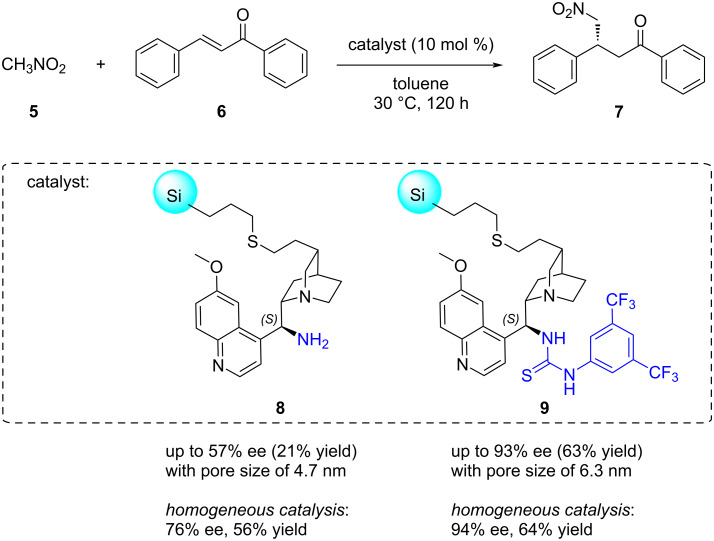
Using confinement of organocatalytic units for improving the enantioselectivity of silica-supported organocatalysts in the Michael addition of nitromethane (**5**) to chalcone (**6**).

By adjusting the pore size of the support, the catalyst selectivity can be influenced. To counteract the decrease in selectivity caused by catalyst immobilisation, the concept of “confinement” was introduced [62], which involves forcing the catalytic moieties into confined spaces. The confinement of the heterogeneous version of cinchona amine and thiourea catalysts was reported, leading to improved enantioselectivity values in the Michael addition of nitromethane (**5**) to chalcone (**6**) through modification of the pore size of mesoporous silica **8** or **9** (Scheme 2) [63]. Thus, for cinchona thiourea, enantioselectivity not only increased with reduced pore size but even reached the level of the homogeneous catalyst when the support pore size was reduced to 6.3 nm. This resulted in obtaining the (*R*)-configured product **7** with 63% yield and 93% ee, as opposed to a 55% yield and 39% ee for a pore size of 11.3 nm [64].

Catalyst supports must meet certain criteria, including being chemically inert, and the supported catalyst should exhibit high stability across various reaction conditions while also being easily recyclable [8,64]. When the solid support is not inert, it can lead to a decrease in selectivity.

**Scheme 3:**
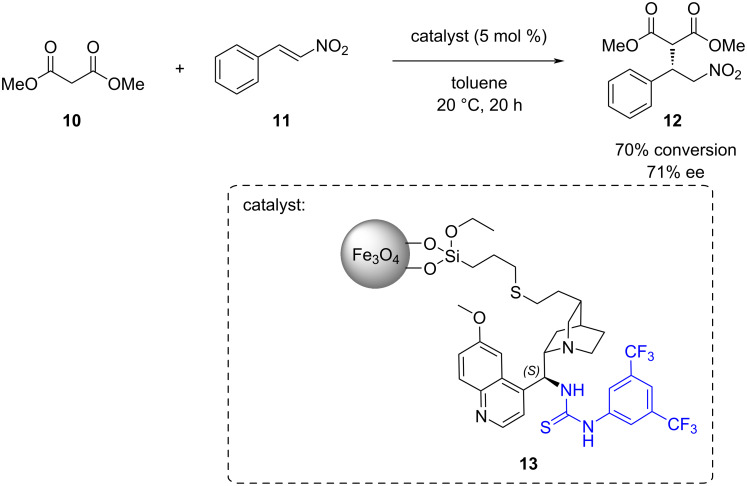
Michael addition catalysed by cinchona thiourea immobilised on magnetic nanoparticles (**13**).

Connon and co-workers have attached a cinchona thiourea organocatalyst to magnetic nanoparticles **13** for the Michael addition of dimethyl malonate (**10)** to *trans*-β-nitrostyrene (**11)** (Scheme 3) [31].

**Scheme 4:**
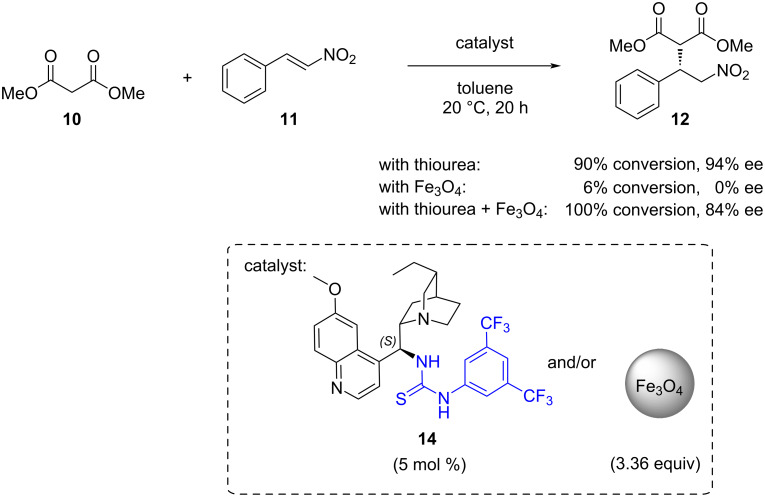
Michael addition catalysed by cinchona thiourea in the presence of magnetic nanoparticles.

To explore the potential impact of nanoparticles on catalyst efficiency, experiments were conducted. It was discovered that the nanoparticles themselves catalysed the formation of the racemic product in the absence of the thiourea catalyst. This finding elucidates the relatively poor enantioselectivity observed in reactions catalysed by the magnetic nanoparticle-supported organocatalyst. To validate this hypothesis, the Michael addition was repeated in the presence of both unsupported thiourea **14** and the nanoparticles. The resulting product was isolated with only 84% ee, indicating that the nanoparticles compete with the thiourea catalyst **14** for the substrate under these conditions (Scheme 4).

**Scheme 5:**
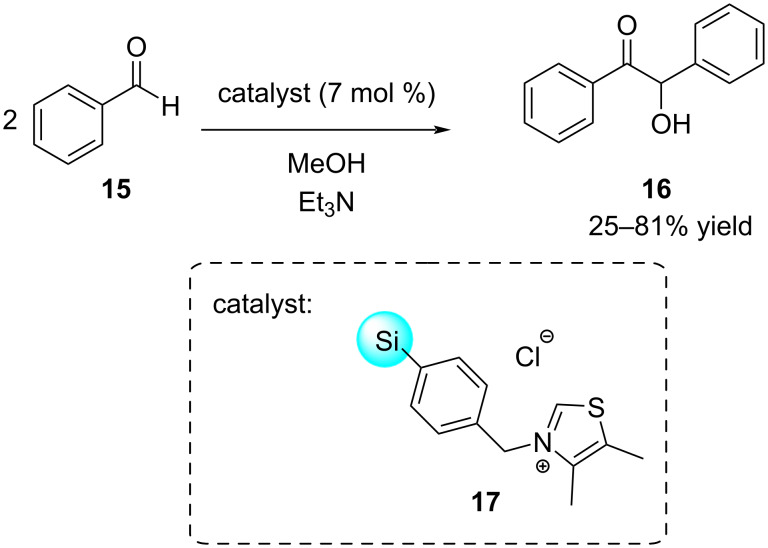
Benzoin condensation catalysed by *N*-benzylthiazolium salt attached to mesoporous material.

Thiel and co-workers examined *N*-benzylthiazolium salts **17** anchored covalently to mesoporous materials in a benzoin condensation reaction (Scheme 5) [65]. Initially good yields were observed, even after a short reaction time, but a drop in yield was seen after reusing the catalysts for a second run. This decrease was attributed to the use of a protic solvent, MeOH, in combination with basic Et_3_N, which could degrade the surface of the support and result in the leaching of the active sites or the restructuring of the mesoporous material. This could have been avoided by performing reactions in a less protic and less polar solvent.

**Scheme 6:**
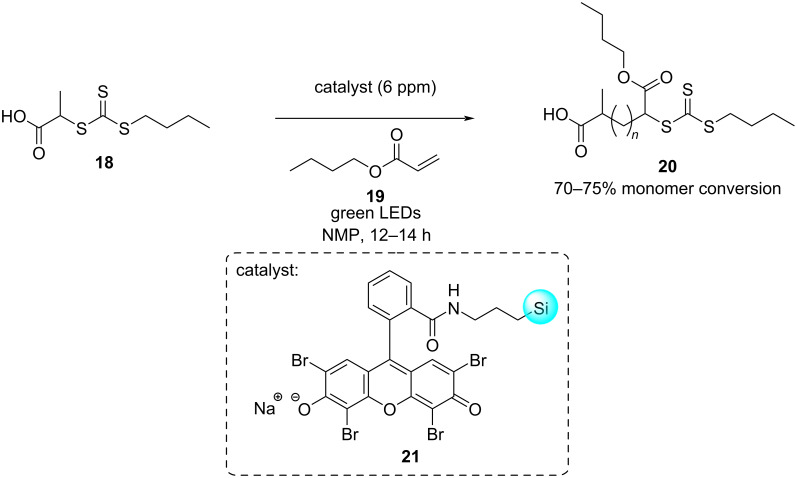
Photoinduced RAFT polymerisation of *n*-butyl acrylate (**19**) catalysed by silica nanoparticle-supported eosin Y **21**, which could be recycled over five reaction cycles.

Attaching catalysts to solid supports also offers the potential for enhancing catalyst stability. Boyer and co-workers reported the use of silica nanoparticle-supported eosin Y **21** as a photocatalyst in reversible addition fragmentation chain transfer (RAFT) photo-polymerisation reactions (Scheme 6) [24]. Previous endeavours utilising the homogeneous catalyst led to catalyst degradation or failure to remove the catalyst properly which resulted in the degradation of the polymer itself [66]. By employing the supported photocatalyst, reduced contamination was demonstrated in the final product and the catalyst could be recycled over five polymerisation cycles at ultralow catalyst loadings (6 ppm), thereby confirming the stability of the catalyst.

### Immobilisation methods

Catalysts are generally immobilised via various interactions between the support and the active catalytic species. These methods can be classified into four categories: covalent bonding, non-covalent interactions (physisorption), ionic bonding, and encapsulation. The catalysts are connected to the support via strong chemical bonds in covalent bonding. In non-covalent interactions, the catalysts are adsorbed onto the support surface through weaker intermolecular forces, such as van der Waals forces and hydrogen bonding. Ionic catalysts form ionic bonds or those that can be ionised under immobilisation conditions. Encapsulation involves physically trapping the catalyst within the pores or cavities of the support [8,67].

Adsorption is a non-covalent reversible technique for attaching organocatalysts to supports. It offers a facile and modular construction of immobilised chiral catalysts with maintained or even improved activity and stereoselectivity [68–70]. The main advantages of catalyst immobilisation by adsorption are that minimal modifications of the parent catalysts are required, few reagents are needed, and it is a relatively cheap and easily implemented method [69,71]. Therefore, adsorption is a popular method for immobilising catalysts. However, this method has a major drawback as the catalyst can easily leach into the solution as it reaches equilibrium between the absorbed species on the surface and the solubilised species. To improve the stability of the supported catalyst, it is important to modify the catalyst and support to enable hydrogen bonding.

This method can also bring the catalyst closer to the support, impacting electronic properties and ligand conformation. With large catalyst molecules, the pores of an ordered mesoporous material being similar in size to the catalyst can create significant diffusion barriers as the catalyst attempts to enter the pores. As a result, the pore channels that are distant from the pore openings are unlikely to be accessible to the catalyst [72].

Ionic bonding is a straightforward and economical immobilisation method. This form of non-covalent immobilisation can be reversed by altering the temperature and ionic strength [71]. Furthermore, the electrostatic interaction between the homogeneous catalyst and the support is robust enough to reduce leaching significantly [67,73]. However, a potential drawback of this technique is that the presence of a charged support can lead to complications, such as distortion of the catalyst structure and changes in the reaction kinetics.

The non-covalent immobilisation of chiral organocatalysts can also be carried out within deep eutectic solvents (DESs). Very recently, a cinchonidine-squaramide organocatalyst was immobilised in three types of natural DESs, namely betaine/sorbitol/water, betaine/xylitol/water, and betaine/mannitol/water [74]. In these systems, the recyclability of the organocatalyst was investigated and several reaction cycles were performed using the same DESs and organocatalyst. For example, in the Michael addition of methyl 2-oxocyclopentane-1-carboxylate and *trans*-β-nitrostyrene, the recyclability of the organocatalyst in the betaine/sorbitol/water DES system was demonstrated up to 10 cycles without any significant decrease in yield (up to 99%) or stereoselectivity (up to 96% ee).

Encapsulation is an irreversible method and the only catalyst immobilisation process that does not require any interaction between the catalyst and the support. Because of this, it is the sole technique which attempts to mimic the homogeneously catalysed reaction process [75]. It typically results in enhanced properties, e.g., augmented morphological stability, tailored physicochemical permeability, and reduced catalyst leakage [76].

When constructing a support around a catalyst, the catalyst must remain stable under the synthesis conditions of the support. If the catalyst can be easily synthesised in a few steps, assembling it within the pores is preferable. However, if the catalyst is difficult to synthesise but remains stable, forming the support around it is advisable [67]. Another limitation of the encapsulation method is that the size of the pore openings in the support must be smaller than the kinetic size of the immobilised catalyst [77].

Covalent tethering is a method of bonding that creates stable catalysts and minimises catalyst leaching. However, this technique has some limitations. Some covalent immobilisation methods involve complex synthetic manipulations, making them unsuitable for large-scale preparations. Furthermore, catalysts bound to carriers may experience restrictions in mobility, limiting their ability to undergo conformational changes necessary for catalysis, especially in the case of enzymes [72,78]. Overall, covalent tethering techniques are the preferred approach to designing stable heterogeneous organocatalysts, provided that the covalent modification does not involve complex synthetic steps.

Recent advancements in materials synthesis and nanotechnology have expanded the repertoire of techniques available for designing catalysts with controlled structures to promote complex reactions selectively. A recent review of Francuisco Zaera [79] discusses key research directions in the transition from homogeneous to heterogeneous catalysis. Special nanostructures, the so-called metal-organic frameworks (MOF), covalent organic frameworks (COF), porous organic frameworks (POF), and hyperbranched systems are formed with a special case of tethering. The ability to predesign both primary and high-order structures serves as a great advantage in these catalytic systems, making them easily fine tuneable. Catalytically active sites can be formed by direct condensation or post-synthetic modifications. In the case of catalyst immobilisation post-synthetic modifications play a more important role.

MOFs are a type of porous crystalline polymers where organic ligands are coordinated to metal clusters [80–90]. The framework can be post-synthetically modified by functional organic sites, often specifically chiral functionalities, giving easily recyclable asymmetric catalysts. The asymmetric sites can be different types, commonly they are binaphthyl-, biphenyl- [91–93] and proline-based [94–95].

COFs are a type of crystalline porous material, consisting of covalently linked organic ligands [96–97]. Since the framework only has organic building blocks, both condensation [98] and post-synthetic modification [99] methods can be used to immobilise organocatalysts. Asymmetric organocatalysis is commonly achieved by pyrrolidone ligands, with great results in a variety of reactions, such as Michael [100] and aldol [101] reactions.

POFs [102] are hydrocarbon systems that contain pores, of which COFs are a subgroup. POFs are widely applied in the fields of gas adsorption and storage, the separation of gases, catalysis, energy storage, photocatalysis, etc., and have many different types, such as hyper-cross-linked polymers (HCPs), polymers of intrinsic microporosity (PIMs), covalent organic frameworks (COFs), extrinsic porous molecules, and porous organic cages [103] etc. Since in terms of organocatalyst immobilisation COFs are the most important, further discussion of POFs is not included in this review.

Hyperbranched systems [104–106] and dendrimers [107–109] have also emerged as alternative soluble supports for catalyst immobilisation. In these systems the catalyst moieties can be built in at the core, at the periphery, or at intermediate positions, affecting the catalytic performances differently [110]. An advantage of dendrimer-supported organocatalysts are their enzyme-like properties [111–112]. Selective binding and cooperative catalysis can give the catalyst high selectivity and activity.

### Interactions between the support and other components

The interaction between the solid support and the organocatalyst [113], reactants [114], product [115] or solvent [116] can significantly impact catalytic activity. These interactions often manifest in various forms of adsorption: physisorption, involving forces like van der Waals interactions and hydrogen bonding, or chemisorption, which may involve ionic or covalent bonding. These adsorptive interactions can alter the electronic properties and conformation of the supported organocatalyst, thereby influencing its catalytic activity compared to its homogeneous counterpart. For instance, Fotaras et al. showed that the incorporation of a tripeptide-like prolinamide-thiourea organocatalyst onto commercially available resins (JandaJel, polystyrene-divinylbenzene, and ChemMatrix) results in diminished catalytic activity, both in terms of yield and enantiomeric excess values, compared to the homogeneous analogue [117]. On the contrary, Dumesic and co-workers showed that the activity of a difunctional organocatalyst in lactose hydrolysis was improved 5.2-fold by immobilisation on different solid supports that mimic the active site channels of enzymes [113].

In a solid-supported system, the solvent can exert a different influence on the catalytic activity compared to a homogeneous catalyst. Solvent molecules present on the support surface might partially obstruct the active sites of the catalyst. Therefore, selecting the appropriate solvent may require adjustments to optimise the performance of the solid-supported organocatalyst [116]. Moreover, before the immobilisation of catalysts, it is necessary to consider whether the solid support itself can catalyse the desired or side reactions. This could be advantageous in some cases, i.e., when the solid support cooperatively helps the reaction [118], but in asymmetric synthesis, background activity of the solid support can lead to lower stereoselectivity, as previously shown in Scheme 4.

### Solutions for limitations of solid-supported organocatalysts

The properties and applicability of an immobilised catalyst depend not only on the immobilisation method and the physicochemical characteristics, porosity, and dimensions of the support, but can also be influenced by several other factors. In a homogeneous system, rapid diffusion, and creation of catalyst–reactant interactions are possible because the organocatalyst is dissolved in the reaction mixture. However, in a solid-supported organocatalyst, the reactants need to diffuse to the active sites on the solid support. Diffusion limitations can decrease the effective concentration of reactants at the catalytic sites, resulting in lower reaction rates compared to the homogeneous catalyst. Thus, optimising reactor design, including appropriate mixing and flow characteristics, can help to minimise these limitations. Higher reactant concentrations may also be necessary to overcome diffusion limitations and maintain suitable concentrations at the active sites [119].

**Scheme 7:**
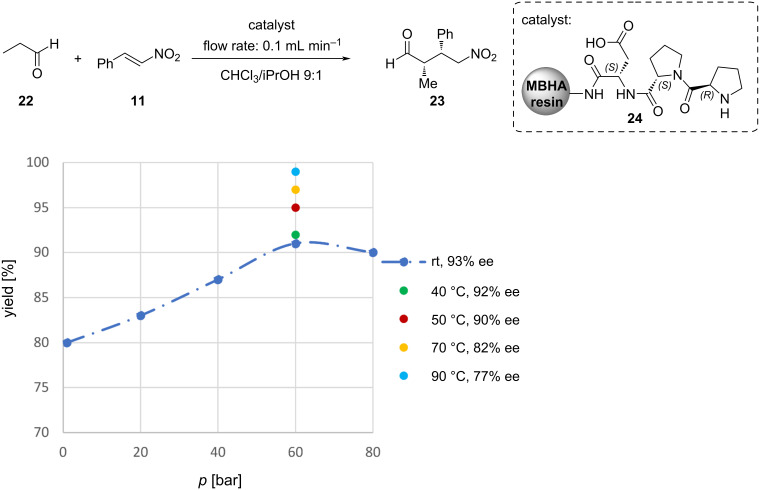
Pressure and temperature dependence of the 1,4-addition of propanal to *trans*-β-nitrostyrene under continuous flow conditions.

Fülöp and co-workers proved the diffusion dependence by employing the Koros–Nowak criterion test [120] in the conjugate addition of propanal (**22**) and *trans*-β-nitrostyrene (**11**) catalysed by a simple solid-supported peptidic catalyst **24** using a continuous flow reactor. To overcome the diffusion limitations, elevated pressure was applied. Increasing the pressure from atmospheric to 60 bar resulted in a 12% increase in yields, however, further increase in pressure proved to be non-beneficial. Moreover, increasing the temperature provided higher yields at optimised pressure (60 bar) but also led to lowered enantioselectivity (Scheme 7) [121].

Zhang and co-workers prepared a novel polymer with an ordered mesoporous system, resulting in a high surface area and uniform pore size. As a result, a high degree of dispersion of the piperazine active sites was achieved, leading to a catalyst exhibiting comparable activity and selectivity to that of a homogenous organocatalyst. Moreover, the hydrophobic surface of the catalyst enhanced the mass transport of organic reactants in water [122]. Based on similar mesoporous catalysts [123–124], it can be concluded that with a properly designed pore size resulting in a high density of active catalytic sites, diffusion control could be diminished.

Furthermore, the solid support may impose restrictions on access to the active sites of the organocatalyst. If the active sites are too close to the support backbone or situated within the porous structure of the support, they are less accessible for larger reactant molecules, thereby affecting the catalytic activity and selectivity. Proper design of the linker between the catalytic sites and the support surface, along with careful selection of the solid support, can help to optimise the accessibility of the active sites and mitigate this limitation [65,125].

Consequently, the properties of the supported organocatalyst also depend on the density of catalytic sites on the support surface, as well as the nature and length of the linker [8]. Additionally, the linker itself could serve as a competitive active site. In the case of enantioselective catalysis, the linker could promote the formation of racemic products, leading to lower stereoselectivity. Moreover, in catalyst co-polymerisation, the organocatalyst might be enclosed within the inner part of the polymer bead, rendering it inaccessible for reactants, or the repeating unit of the polymer could act as a competitive active site.

**Scheme 8:**
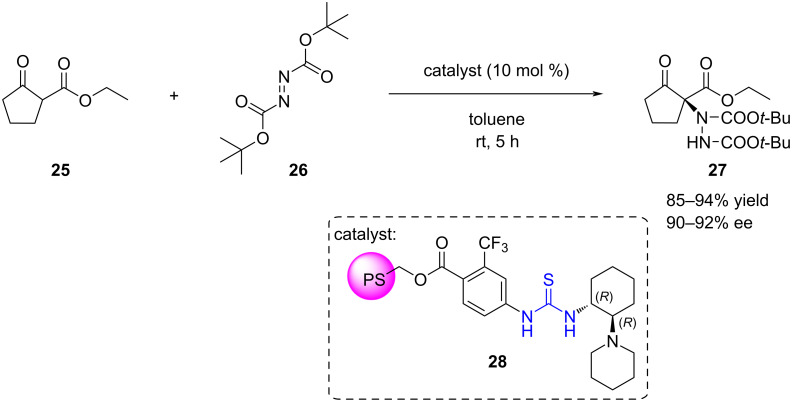
α-Amination of ethyl 2-oxocyclopentanecarboxylate catalysed by PS-THU which could be recycled over 9 reaction cycles.

On the other hand, in some cases, the support surface can even impede unwanted side reactions. Pericàs and co-workers immobilised a thiourea organocatalyst on PS (**28**) and applied it in the enantioselective α-amination of 1,3-dicarbonyl compounds [36]. Unlike homogeneous thioureas, catalyst **28** is not irreversibly deactivated by azodicarboxylate reagents. In the attempt to recycle catalyst **28** for the reaction between ethyl 2-oxocyclopentanecarboxylate (**25**) and di-*tert*-butyl azodicarboxylate (**26**) (Scheme 8), a decline in catalytic activity was initially noticed. While this could be attributed to the nucleophilic deactivation of thioureas by azodicarboxylates in a homogeneous phase, as noted by Takemoto [126], it was suggested that this interaction would be significantly impeded by the polymer backbone. Alternatively, it was proposed that this degradation could be due to protonation of the basic tertiary amine unit. It was found that washing the resin with triethylamine between reaction cycles was sufficient to regenerate the catalyst. Consequently, high yields (85–94%) and enantiomeric excess values (90–92%) were consistently achieved over 9 reaction cycles.

**Scheme 9:**
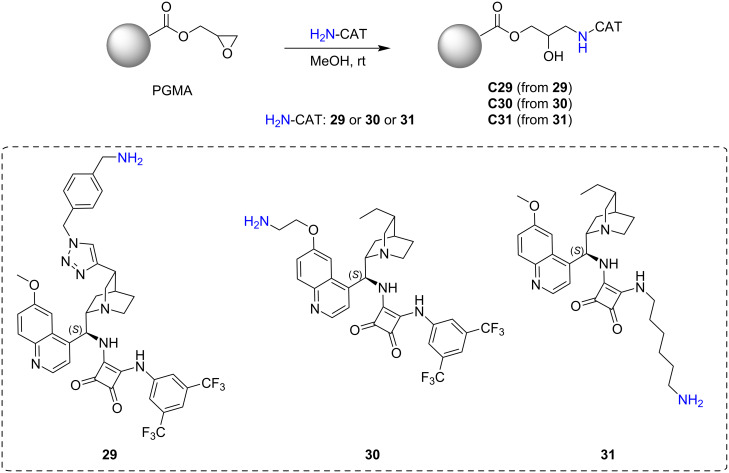
Preparation of supported catalysts **C29–C31** from cinchona squaramides **29–31** modified with a primary amino group.

In a previous work conducted in our research group, we investigated the influence of various linkers of cinchona squaramide organocatalysts immobilised on a poly(glycidyl methacrylate) (PGMA) solid support [127]. The support consisted of well-defined monodispersed PGMA microspheres, which were prepared through thorough parameter optimisation. Three amine-functionalised cinchona derivatives **29–31** were immobilised on this polymer support by utilising its reactive epoxy groups (Scheme 9).

**Scheme 10:**
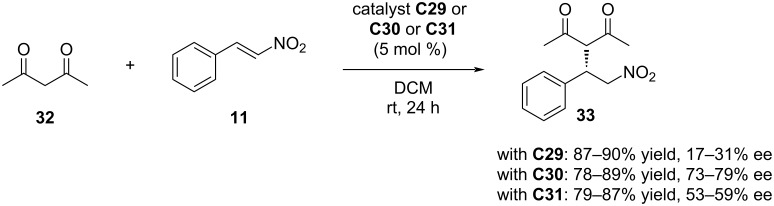
Application of PGMA-supported organocatalysts **C29–C31** in the asymmetric Michael addition of pentane-2,4-dione (**32**) and *trans*-β-nitrostyrene (**11**).

These structurally diverse precatalysts were prepared by modifying the cinchona skeleton at different positions to investigate how the amino group-containing linker affects them. The immobilised organocatalysts' catalytic activities and enantioselectivity values were evaluated in the Michael addition of pentane-2,4-dione (**32**) and *trans-*β-nitrostyrene (**11**). The catalysts could be reused over five reaction cycles through centrifugation, without significant loss of activity (Scheme 10).

The catalysts showed no significant differences in terms of yields. However, catalysts **C30** and **C31** achieved higher enantiomeric excess values (up to 79% ee and 59% ee, respectively) compared to catalyst **C29** (up to 31% ee). Among the immobilised catalysts, **C30** produced the best results. Catalyst **C29**, which showed lower selectivity, features a 1,2,3-triazole-4-yl unit as the substituent at the tertiary amine-containing quinuclidine motif, whereas **C30** and **C31** have an ethyl group attached to the ring in this position. Additionally, catalyst **C31** has a longer-chain linker, but its squaramide NH groups are more acidic due to binding with an electron-withdrawing group. This acidity can result in stronger hydrogen bonds between the substrate and the catalyst **C30**, which contains a bis(trifluoromethyl)phenyl-modified squaramide moiety. This stronger interaction potentially enhances the catalyst–substrate interaction, allowing for more precise stereocontrol of the reaction [128–132]. Therefore, the difference in enantioselectivity values may be attributed to these electronic effects. Ultimately, the most favourable outcomes were achieved with catalyst **C30** at 0 °C, with yields reaching up to 84% and enantiomeric excess reaching 96%.

**Scheme 11:**
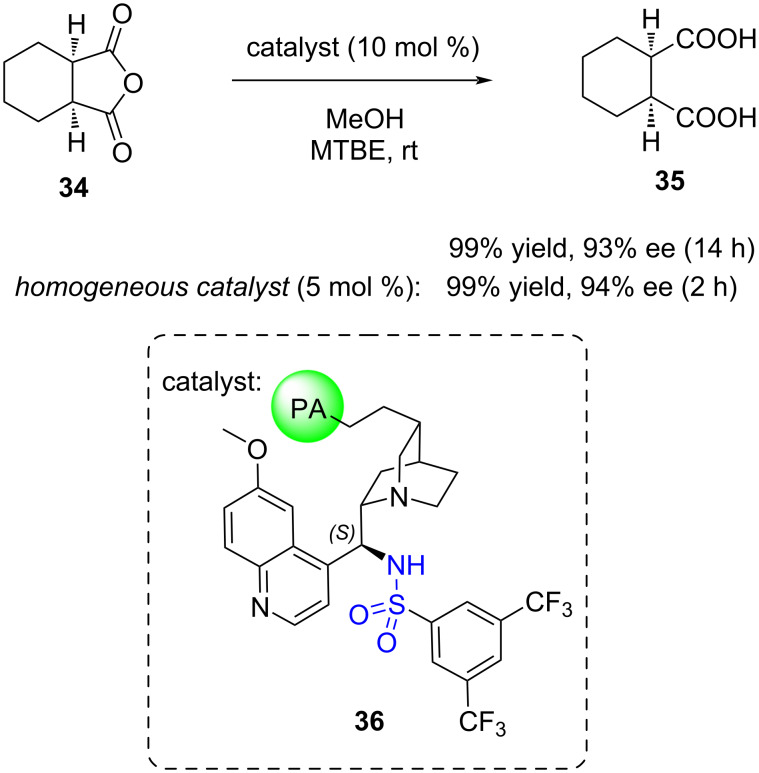
Alcoholytic desymmetrisation of a cyclic anhydride **34** catalysed by polyamide-supported cinchona sulfonamide **36**.

As highlighted in this review, different factors can aid in the design and optimisation of solid-supported organocatalysts, ensuring their catalytic activity is comparable or even superior to their homogeneous counterparts. A notable example of a highly recyclable solid-supported organocatalyst was demonstrated by List and co-workers, who showcased the robustness of a cinchona alkaloid-based sulfonamide organotextile catalyst **36** (immobilised on nylon 6,6) through hundreds of recycling experiments (Scheme 11) [15]. The organotextile catalyst **36** exhibited a very similar enantioselectivity (93% ee) to the homogeneous catalyst (94% ee) in the alcoholytic desymmetrisation of a cyclic anhydride **34** albeit requiring a slightly longer reaction time. Compared to polymer films, textile fibres offer a significantly higher surface area, potentially contributing to the maintenance of high enantioselectivity (>90% ee) for over 250 cycles.

## Conclusion

Solid-supported organocatalysts can provide an environmentally friendly and economical solution even for industrial processes. To maximise their adoption by industry, efficient catalysts that can be easily recycled need to be developed. Immobilisation on solid supports such as polymers, silica, glass beads and magnetic nanoparticles has proven effective, although each method presents unique challenges regarding catalytic activity, selectivity and stability. A number of factors need to be taken into account during the development process. The surface area, morphology and pore size of the solid support have a strong influence on the catalytic properties. Innovative approaches such as confinement effects and advanced material designs such as MOFs, COFs and hyperbranched systems offer promising solutions to enhance catalyst efficiency and selectivity. It is also crucial that the supports meet certain requirements: they must be chemically inert, stable and easily recyclable. Examples can also be found where the stability of the catalyst is increased through the attachment to the support.

Of the immobilisation methods, covalent tethering is preferred because of the resulting stable immobilisation. However, simple catalyst grafting with few steps is important and favourable for industrial applications. Interactions between the support, the catalytically active site and other components must also be taken into account in catalyst development. Additionally, the selection of the appropriate solvent is critical.

To mitigate diffusion limitation, it is important to ensure appropriate mixing and flow characteristics and adequate concentration of reactants and catalytic units. Flow chemistry can be easily combined with solid-supported organocatalysis, while even elevated pressure can be applied to reduce diffusion limitations. The size, rigidity and character of the linker also play an important role in catalyst design.

Despite these advances, the development of highly efficient and recyclable organocatalysts remains a challenge. Trade-offs between immobilisation methods and the catalytic performance require further research. Future directions include improving immobilisation strategies, exploring new support materials and optimising reaction conditions to alleviate diffusion limitations and improve the availability of active sites.

In summary, the evolving development of solid-supported organocatalysts has significant potential for industrial applications, particularly in the pharmaceutical and fine chemical industry. The design of recyclable, robust and high-performance organocatalyst systems will continue to encourage innovation in this area, contributing to more sustainable and efficient catalytic processes.

## Data Availability

Data sharing is not applicable as no new data was generated or analyzed in this study.
